# Recognize and Alleviate a Resource Management Conundrum Facing Science Diaspora Networks

**DOI:** 10.3389/frma.2022.898770

**Published:** 2022-06-28

**Authors:** Dorothy L. Butler, Barbara Del Castello, Steve Elliott, Elana R. Goldenkoff, Isabel A. Warner, Alessandra C. Zimmermann

**Affiliations:** Science Diplomacy Exchange and Learning, National Science Policy Network, San Francisco, CA, United States

**Keywords:** knowledge network, resource management, science diplomacy, organizational analysis, brain circulation, network approach model, bounded rationality

## Abstract

Increasingly, science diaspora networks are managed by formal organizations such as embassies or non-profit organizations. Researchers have studied these networks to understand how they influence international collaborations and science diplomacy, and to determine which network activities foster those outcomes and which do not. In this perspective, we suggest that many of these network organizations confront an underappreciated conundrum for managing resources: organizations with few resources must learn how to obtain more resources despite lacking means to do so. To substantiate our suggestion, we do the following. We review exploratory results from a study of network organizations that indicate that these organizations generally lack resources, learn too little from each other, and struggle to overcome the resource conundrum. We also show that this conundrum is expected from organizational theory based on bounded rationality. To help organizations confront the issue, we do the following. First we provide a new database of operating science diaspora networks. We encourage managers of network organizations to use it as a resource to identify peers with whom to regularly exchange knowledge about securing resources. We also suggest that other scientific organizations should infuse network organizations with fresh resources. Ultimately, we urge all relevant stakeholders to recognize that the conundrum results not from the shortcomings of individual managers, but rather is a legitimate organizational phenomena that must be addressed by organizational design.

## Introduction

Highly educated and skilled researchers in STEM (science, technology, engineering, and math) increasingly emigrate from their countries of origin to pursue educational and career opportunities elsewhere (Anand et al., 2009; De Domenico et al., 2016; Netz et al., 2020). Often these individuals are described as living in diasporas (Barré et al., 2003; Séguin et al., 2006; Meyer, 2019). Researchers use the terms “diaspora knowledge networks” and “scientific diaspora networks” to describe the social, economic, and political groups that have formed to link these transnational and migrant populations of professional and scientific communities (Meyer, 2001; Brown, 2002; Barré et al., 2003; Mahroum et al., 2006).

Increasingly, diaspora networks are managed by formal organizations (Gamlen, 2014; Gamlen et al., 2019). Sometimes countries create organizations within their foreign embassies and consulates to manage a network in a host country. For instance, the Office of Science and Technology Austria, Washington (OSTA) is a department of the Austrian Embassy to the United States. OSTA manages its Research and Innovation Network Austria, comprised of Austrian researchers across North America. Sometimes international organizations create subunits to connect networks of members. For instance, the Marie Curie Alumni Network has 33 local chapters to connect current and previous recipients of Marie Skłodowska-Curie Actions funding. Sometimes emigres in a host country create non-governmental organizations to provide bank accounts, listservs, bylaws, and managerial roles for their network. One example is the Italian Scientists and Scholars in North America Foundation, which connects more than 3,000 primarily Italian researchers who live in North America.

Many of these network organizations confront a resource management conundrum. They work with little or transient budgets, time, and staffing. We recently completed an interview study of managers from 21 organizations (Butler et al., 2022). Our results indicate that managers recognize this resource scarcity, that they want to improve their situations, and that they often cannot begin this process due to lack of resources. The conundrum for organizations with few resources is to learn how to obtain more resources despite lacking the means to do so.

In this Perspective, we argue that this conundrum should be recognized, further studied, and alleviated as a legitimate organizational phenomenon confronting diaspora network organizations. Our strategy is as follows. First, we provide a new database of extant science diaspora networks to update previously published lists (Meyer and Brown, 1999; Brown, 2002; Meyer and Wattiaux, 2006). The database is a resource for further studies of network organizations. Next, we overview exploratory results from our recent work indicating that network organizations confront the resource conundrum (Butler et al., 2022). We then show that the conundrum is expected from organizational theory. We close with suggestions for how different kinds of stakeholders could help alleviate the conundrum.

## A Database of Diaspora Network Organizations

We define a diaspora network organization as a formal organization that manages a diaspora network. A diaspora network connects people of a particular national or regional background in a host country or region. A network organization has at least one person who manages the budget, activities, communications, and resources for the network. Commonly they have bylaws and can be subunits of parent organizations (e.g., of embassies), non-governmental organizations, clubs, etc. Network organizations for science in particular aggregate scientists for the purposes of professional development, collaboration, or community building (Brown, 2002; Burns, 2013; Tejada, 2013; Bonilla et al., 2018).

We provide here an updated database of active science diaspora network organizations, with links to their websites (see Supplementary Table 1). This database updates similar efforts published earlier (e.g., Meyer and Brown, 1999; Brown, 2002; Meyer and Wattiaux, 2006; Echeverría-King et al., 2022). For our recent study, we created an initial list of active networks and continued to iterate it after the study was completed (Butler et al., 2022). We have found 49 active networks through literature reviews, web searches, and word of mouth, and there are likely many more in existence that were not captured by our study.

Table 1 summarizes the geographical, structural, and lifespan diversity of the networks. All authors reviewed networks' websites and unanimously grouped networks into categories by Region, Structure, and Age. Compared to other regions, there is a disproportionately large number of networks with ties to European countries in the database, for which we suggest two potential explanations. First, there may be collection bias by our North American-based research team, as there are many European networks in North America. Second, the disproportions may reflect real disparity in the prevalence of diaspora networks across regions, with those from or in high income countries in the global north more able to develop networks compared to those from or in low and middle income countries. We encourage further iterations of the database to capture more networks across more regions. We also encourage further research to investigate the reality and causes of this apparent regional disparity.

**Table 1:** Summary of science diaspora networks.

**Region (49)**			**Organization structure (49)**	
	Region of origin	Host region	Volunteer*	8 (16.3%)
Africa	0 (0.0%)	0 (0.0%)	NGO**	27 (55.1%)
Asia^∧^	10 (20.4%)	5 (10.2%)	Govt.***	14 (28.6%)
Europe	25 (51.0%)	3 (6.1%)	**Age of the organization (42)**	
Latin America	8 (16.3%)	1 (2.0%)	>20 years	17 (40.5%)
North America^∧∧^	0 (0.0%)	23 (46.9%)	19–10 years	12 (28.6%)
SWANA^∧∧∧^	3 (6.1%)	0 (0.0%)	10–5 years	8 (19.1%)
World	3 (6.1%)	17 (34.7%)	<5 years	5 (11.9%)

*All percentages are computed in relation to the number of organizations represented in parentheses. Compared to Region and Organization Structure, Age of Organization summarizes fewer total organizations due to inability to determine ages for seven organizations*.

*^∧^Includes Australasia*.

*^∧∧^Excluding countries commonly classed in Latin America*.

*^∧∧∧^South West Asia/North Africa*.

^*^Volunteer indicates organizations that note no larger organization governing them and no not-for-profit status identified.

^**^NGO or non-governmental organizations, are organizations with not-for-profit status or that were founded as part of a parent NGO.

^***^Govt. or governmental groups founded are those founded by federal directive or under the purview of a federal body.

## Corroboration of the Conundrum

Between January 2021 and March 2022, we conducted an exploratory study of scientific diasporas networks and those who manage them. We interviewed managers from a sample of 21 networks, which varied in network size, type of organization, host countries, countries of origin, aims, etc. We asked them to characterize how they conceptualize network success, network relevance to diplomacy, current challenges, and future plans. Full methods and results can be found in our recent report (Butler et al., 2022). Here we describe the resource management conundrum common to many diaspora networks. We found this sentiment existed across all three inductively-coded themes of Challenges, Success, and Future.

The conundrum for organizations with few resources is to learn how to secure more while lacking resources to do so. One of the most basic ways managers can do this is to regularly interact with other managers from peer organizations to share operational knowledge and tactics. Responses to our interview questions indicated that many managers struggled to find resources, especially time, to accomplish such tasks.

### Challenges

The majority of interviewees cited few resources as a major challenge to the operation of their networks. In many cases, managers described significant amounts of work completed on minuscule budgets, with little or no infrastructure or staff to support the missions of their network organizations. As a result, managers reported spending their time accomplishing highly programmed tasks that they have experience with, such as managing listservs, organizing events, and addressing member needs. This result was especially common for managers of organizations younger than 5 years. Managers of older organizations noted that they hardly interacted with their peers at similar organizations, and they worried that they were reinventing organizational structures and processes rather than sharing knowledge. A majority of interviewees across all organizations voiced feelings of isolation given no, little, or rarely sustained interactions with managers at peer organizations. Managers with comparatively greater resources, especially funding from parent or outside organizations, reported disconnects between satisfying the needs of the funders and satisfying the needs of the network members. Relatedly, for managers of organizations that were older or had more resources, especially formal embassy connections, they reported being more likely to spend resources on science diplomacy to influence policy.

### Success

Nearly all interviewees conceptualized success for their organizations as making connections, a result consistent with findings of previous studies on diaspora networks' objectives and types of engagement activities hosted (Brown, 2002). The more connections an organization helped foster, the more successful its managers judged the organization. Managers most commonly talked about making connections between the individual members of their particular network, e.g., to foster friendships, research collaborations, and mentor/mentee relationships. They also discussed connections between network members and representatives from scientific or governmental organizations (e.g., program managers at funding agencies, staffers and diplomats in governments and embassies, and hiring managers at universities, non-profits, or firms). These connections then fostered the professional development of network members. Fewer than five interviewees discussed making connections with peer managers at other network organizations as markers of success.

### Future

When asked about the future of their networks, most interviewees expressed desires to increase and strengthen connections, most commonly with their members, and sometimes with relevant governments from countries of origin to increase their influence and acquire more support. Interviewees recognized the impact they could have by working with other networks, especially as new global scientific challenges arise, and were interested in sharing operational knowledge with their peers. Many groups asserted that they wanted to form inter-network connections in the future.

Altogether, these results indicate that managers are acutely aware of their resource scarcity, and that they can rarely spend their resources to learn organizational processes from their peers. The results also indicate that there are opportunities for these organizations to help alleviate this problem. They already characterize organizational success as fostering connections, a criterion that can be extended to include explicitly making connections with peer managers in other networks to learn organizational processes for, among other things, securing resources. Furthermore, if these groups more regularly shared information with each other, they could strengthen their diplomatic advocacy and ease burdens of having to individually create structures and techniques to address similar issues.

These results are exploratory and suggestive, and further studies are needed to test the extent to which network organizations confront the resource management conundrum and to assess the scope and impacts of the conundrum. We next suggest that such efforts would prove fruitful, as the conundrum is expected from organizational theory.

## The Conundrum From Theory

As a general organizational phenomenon, the resource management conundrum is a consequence of organizational theory based on bounded rationality (Simon, 1991, 1997; March and Simon, 1993). In this theory, people and organizations are agents of bounded rationality and thus have imperfect knowledge of the world. They curtail their searches for solution to problems when they have developed satisfactory—rather than optimal—solutions, and they develop standard procedures, routines, or programs to deal with recurring problems. The theory of bounded rationality is widely used to characterize human and organizational learning in fields such as organizational studies, economics, cognitive psychology, and political science (Jones, 1999; Wheeler, 2018).

The conundrum depends on two regularities of the theory related to how organizations learn new procedures. First, the more an organization is dissatisfied with its procedures to address a need, the more likely it is to search for better procedures (March and Simon, 1993, 194). It is often beneficial to search similar but distinct organizations, as doing so often requires fewer resources than developing and testing new procedures *de novo*. Second, managers tend to spend resources on procedures that are highly programmed and not on those that are highly unprogrammed (March and Simon, 1993, 206–207).

An organization is more likely to get one of two outcomes from search and learning processes depending on the amount of its current resources, which can be mediated by the quality of its current search procedures. The more resources an organization begins with, the more probable it is to achieve the Getting Richer Outcome. The fewer resources an organization begins with, the more probable it is to achieve the Staying the Same Outcome.

Getting Richer Outcome: If an organization already has resources to search similar organizations for alternative procedures to secure resources, then it can learn from other organizations. The learning process will be more or less efficient depending on the organization's store of highly-programmed search and learning processes, which enable more efficient use of resources for search and learning. If an organization lacks good search and learning procedures, but it has surplus resources, it will spend some of that surplus on highly-unprogrammed, likely inefficient, searches. In either situation, the organization increases its chances to find better alternatives to its current procedures for securing resources.

Staying the Same Outcome: If an organization lacks resources to search and learn from other organizations, then it cannot search and learn from other organizations. Even if it has highly programmed and effective search procedures, but not the resources to use them, they are of no practical use to the organization. And if an organization lacks good search procedures and resources, with no resources it cannot develop better procedures or perform even inefficient searches. In either situation, the organization cannot learn from other similar organizations. It will likely persist at its current resource level.

The conundrum for organizations with few resources and a desire to secure more resources is to learn how to avoid the Staying the Same Outcome. Alleviating the conundrum is neither a trivial nor an obvious task, nor does it admit of a single solution.

## Discussion and Recommendations

The network model of researcher diasporas has two functions. First, it describes emigre activity, as a diaspora's members connect to each other *via* regular communication channels (e.g., listservs, social media, etc.) that are characteristic of social networks (Meyer, 2001; Newman, 2003). Second, the model normatively indicates how emigres and their countries of origin and host countries should mutually interact (Mahroum et al., 2006; Tejada, 2013; Tejada et al., 2014; Bonilla et al., 2018; Radwan and Sakr, 2018). For this second function, diaspora networks are termed networks because doing so links them to a model of researcher migration that differs from the brain drain model (Brown, 2002; Séguin et al., 2006; Zong and Lu, 2017). The brain drain model implies that researchers who leave their countries of origin deprive those countries of benefiting from the expertise of the researchers. Conversely, the network model enables countries to treat their researchers abroad and the communities they host as resources for international knowledge exchange, economic development, and diplomacy (Meyer, 2001; Ciumasu, 2010; Burns, 2013).

Our discussion here corroborates but does not prove the expectation from theory that managing organizations for scientific diaspora networks face a resource management conundrum. Further research is needed. Nonetheless, we judge it likely that network organizations and their managers regularly contend with the Staying the Same Outcome.

Below we suggest three general strategies to alleviate the resource conundrum—one applicable to all science diaspora network stakeholders, one for network managers, and another for current and potential partner organizations (e.g., funding bodies, universities, and multilateral institutions). These strategies inform the normative aspect of the network model.

First, all science diaspora network stakeholders should recognize that the Staying the Same Outcome is a legitimate management conundrum. It results from resource constraints—not from manager shortcomings. In our interviews, managers often blamed themselves for not having the time or knowledge of their peers to reach out for ideas on how to secure more resources. Many also mistakenly worried that their organizations were unique in their scarcity of funding, time, and staff. There may also be discomfort in acknowledging to peers a need for ideas on how to successfully secure more resources. We hypothesize that if more people recognize that the conundrum is an expected outcome for organizations with few resources, and not the product of managers' shortcomings, then more people will be willing to discuss it and find strategies to alleviate it.

Next, network managers in particular can usefully turn unprogrammed search and learning activities into more highly programmed activities or routines. Rather than relying on happenstance interactions with their peers at similar organizations, managers would be wise to develop regular and structured practices for consistently exchanging ideas and knowledge about techniques to secure resources. To do so, they must revise their criteria of organizational success to include regular professional development specifically for managers *as managers*. They must also budget and protect some resources, no matter how small, to connect with and learn from their peers at other organizations. Such resources can be as little as 6 hours a year vouchsafed to call peers and talk about operational processes. A key aide to this effort is a list of networks from which peers can be identified (see Supplementary Table 1).

Finally, other organizations could fruitfully infuse network organizations with resources. Diaspora networks do not exist in a vacuum apart from other scientific organizations, with which their goals often align. Many funders, government and philanthropic alike, further science primarily by funding research projects. There are opportunities to fund more capacity-building projects for networks organizations. These projects could provide networks with resources to hire staff, develop and improve regular procedures, and secure larger and longer-term funding from additional funders. Scientist networks are resources for any country they interact with, including host countries. Funders in one country may usefully partner with peer funders in a second country to mutually support networks that span their borders.

Opportunities exist to strengthen diaspora networks and science diplomacy by supporting communities of practice among the managers of network organizations. This position is notably taken by EURAXESS, which focuses on European contexts and enjoys European Union (EU) funding to seed, support, and grow diaspora networks abroad. EURAXESS provides several guides for doing so (e.g., Mahmoud and Bodnarova, 2019). The EU is unique, but it is not the only multilateral organization that could support stronger communities of practice, especially for low- and middle-income countries (Séguin et al., 2006). Similar and additional efforts could be further supported by, for instance, the African Union, Organization of American States, and the United Nations *via* programs like Transfer of Knowledge through Expatriate Nationals (TOKTEN) and The World Academy of Sciences (TWAS). Additionally, as many diasporic scientists are part of the academic labor market, institutions such as the International Association of Universities can play animportant role in supporting the globalization of research and ducation (Welch and Zhen, 2008, Larner, 2015).

Science diaspora networks bring immense value to their members, partners, and diplomacy. An increasing array of actors are noticing that value. Current and future stakeholders should recognize and work to alleviate the management conundrum confronting these networks. Doing so will benefit science and diplomacy.

## Data Availability Statement

The original contributions presented in the study are included in the article/Supplementary Material, further inquiries can be directed to the corresponding author.

## Ethics Statement

Ethical review and approval was not required for the study on human participants in accordance with the local legislation and institutional requirements. Written informed consent for participation was not required for this study in accordance with the national legislation and the institutional requirements.

## Author Contributions

All authors contributed to the conception, design, and conduct of the project. AZ organized the database of networks. SE wrote the first draft of the manuscript. DB, BD, EG, IW, and AZ wrote sections of the manuscript. All authors contributed to manuscript revisions, read, and approved the submitted version.

## Funding

This research received support from the National Science Policy Network.

## Conflict of Interest

The authors declare that the research was conducted in the absence of any commercial or financial relationships that could be construed as a potential conflict of interest.

## Publisher's Note

All claims expressed in this article are solely those of the authors and do not necessarily represent those of their affiliated organizations, or those of the publisher, the editors and the reviewers. Any product that may be evaluated in this article, or claim that may be made by its manufacturer, is not guaranteed or endorsed by the publisher.

## Supplementary Material

Supplementary Table 1 Database of scientific diaspora network organizations.
